# Maternal obesity during pregnancy and cardiovascular development and disease in the offspring

**DOI:** 10.1007/s10654-015-0085-7

**Published:** 2015-09-16

**Authors:** Romy Gaillard

**Affiliations:** The Generation R Study Group (Na 29-15), Erasmus University Medical Center, PO Box 2040, 3000 CA Rotterdam, The Netherlands; Department of Pediatrics, Erasmus University Medical Center, Rotterdam, The Netherlands; Department of Epidemiology, Erasmus University Medical Center, Rotterdam, The Netherlands

**Keywords:** Maternal obesity, Excessive gestational weight gain, Adverse pregnancy outcomes, Fetal death, Childhood obesity, Childhood cardiovascular risk factors, Adult cardiovascular risk factors

## Abstract

Maternal obesity during pregnancy is an important public health problem in Western countries. Currently, obesity prevalence rates in pregnant women are estimated to be as high as 30 %. In addition, approximately 40 % of women gain an excessive amount of weight during pregnancy in Western countries. An accumulating body of evidence suggests a long-term impact of maternal obesity and excessive weight gain during pregnancy on adiposity, cardiovascular and metabolic related health outcomes in the offspring in fetal life, childhood and adulthood. In this review, we discuss results from recent studies, potential underlying mechanisms and challenges for future epidemiological studies.

## Introduction

Obesity, cardiovascular disease and type 2 diabetes are major public health problems. These common diseases have a large impact on morbidity and mortality in the general adult population [1–4]. Various socio-demographic and lifestyle-related risk factors for these diseases have been identified in both childhood and adulthood, of which many have been published in this journal [5–26]. Also, adverse exposures during the fetal and early postnatal period may influence the risk of adverse health outcomes in later life [27]. Previous epidemiological studies have shown that both low and high birth weight are associated with increased risks of obesity, cardiovascular disease and type 2 diabetes in later life [27–31]. These findings are supported by experimental animal studies [27, 32]. Thus, previous research suggests that both restricted and excessive nutritional in utero environments may lead to cardiovascular disease in later life.

In Western countries, maternal obesity during pregnancy is an important adverse risk factor for an excessive nutritional in utero environment [33, 34]. Currently, the obesity prevalence rate in pregnant women is estimated to be as high as 30 % [35, 36]. Also, based on the US Institute of Medicine (IOM) guidelines, approximately 40 % of women gain an excessive amount of weight during pregnancy in Western countries [37]. The IOM guidelines define optimal ranges of maternal weight gain during pregnancy according to a mother’s prepregnancy body mass index, and have been established based on evidence from observational studies that relate gestational weight gain to various maternal and offspring outcomes (Table 1) [37].

**Table 1:** Institute of medicine criteria for gestational weight gain

Recommended gestational weight gain defined according to the Institute of Medicine Criteria	
Prepregnancy body mass index	Recommended amount of total gestational weight gain in kg
Underweight (body mass index <18.5 kg/m^2^)	12.5–18
Normal weight (body mass index ≥18.5–24.9 kg/m^2^)	11.5–16
Overweight (body mass index ≥25.0–29.9 kg/m^2^)	7–11.5
Obesity (body mass index ≥30.0 kg/m^2^)	5–9

Recommended gestational weight gain guidelines according to women’s prepregnancy body mass index. Adapted from the IOM criteria [37]

As described previously, both maternal prepregnancy obesity and excessive gestational weight gain seem to have persistent effects on various childhood outcomes [38]. This review update is focused on the associations of maternal obesity and excessive weight gain during pregnancy with specifically cardiovascular and metabolic development in the offspring from fetal life until adulthood. Results from recent studies, methodological considerations, potential underlying mechanisms and challenges for future studies are discussed.

## Fetal development

Maternal prepregnancy obesity and excessive weight gain during pregnancy are important risk factors for a variety of adverse fetal outcomes (Table 2). A meta-analysis based on published results from 9 observational studies showed that the unadjusted odds ratios of a stillbirth were 1.47 (95 % confidence interval (CI) 1.08–1.94) and 2.07 (95 % CI 1.59–2.74) among overweight and obese pregnant women, respectively, compared with normal-weight pregnant women [39]. When this meta-analysis was restricted to studies that performed adjusted analyses, results did not materially change. In line with this meta-analysis, a study among 1857,822 live single births in Sweden showed that a higher maternal body mass index in early pregnancy was associated with an increased risk of infant mortality, especially among term births (≥37 weeks) [40]. This association was mainly explained by congenital anomalies, birth asphyxia, other neonatal morbidities, sudden infant death syndrome or infections [40]. A meta-analysis among 18 observational studies showed that maternal obesity was associated with an increased risk of a number of congenital anomalies, including neural tube defects, cardiovascular anomalies, cleft palate, hydrocephaly and limb reduction anomalies [41].

**Table 2:** Adverse offspring outcomes of maternal obesity during pregnancy

Fetal outcomes	Childhood outcomes	Adult outcomes
Stillbirth Neonatal death Congenital anomalies Large size for gestational age at birth Neonatal hypoglycemia Referral to neonatal intensive care unit	Obesity Adverse body composition Increased blood pressure Adverse lipid profile Increased inflammatory markers Impaired insulin/glucose homoeostasis	Obesity Increased blood pressure Adverse lipid profile Impaired insulin/glucose homoeostasis Premature mortality

Cardiovascular and metabolic consequences during fetal life, childhood and adulthood of maternal prepregnancy obesity or excessive gestational weight gain

It is well-known that maternal prepregnancy obesity and excessive gestational weight gain are associated with an increased risk of large-size-for gestational age at birth [42, 43]. A recent meta-analysis among 13 studies showed that, as compared to maternal prepregnancy normal weight, maternal prepregnancy obesity was associated with a twofold higher risk of delivering a large size for gestational age infant [42]. Studies using more detailed maternal weight and fetal growth measurements showed that a higher maternal prepregnancy body mass index was associated with a higher estimated fetal weight from second trimester onwards, with stronger associations at older gestational age [44]. Especially higher second and trimester maternal weight gain seem to be associated with an increased risk of large size for gestational age at birth [45, 46]. Furthermore, multiple observational studies have reported that maternal prepregnancy obesity and excessive gestational weight gain are associated with increased risks of low Apgar score, neonatal hypoglycemia and referral to neonatal intensive care unit [47]. Recently, a meta-analysis of multiple randomized controlled trials showed that dietary and physical activity interventions aimed at reducing maternal weight gain during pregnancy may lead to small reductions in the amount of gestational weight gain and to lower risks of adverse fetal outcomes [48].

Fewer studies assessed the direct influence of maternal obesity during pregnancy on placental and fetal cardiovascular and metabolic development. It has been shown that maternal prepregnancy obesity is associated with higher placental weight, placental vascular dysfunction, placental inflammation and alterations in placental transporters activity and mitochondrial activity [49–55]. A study among 1035 mother-infant pairs showed that placental weight partially mediates the effect of maternal prepregnancy obesity and excessive gestational weight gain on birth weight [49]. Higher maternal prepregnancy body mass index and gestational weight gain have been associated with higher leptin and C-peptide levels in cord blood [56–59]. The associations of maternal body mass index and weight gain with detailed embryonic and fetal cardiovascular development remain to be studied.

Thus, both maternal prepregnancy obesity and excessive gestational weight gain lead to increased risks of adverse fetal outcomes. Overall, maternal prepregnancy obesity appears to be more strongly associated with adverse fetal outcomes than excessive maternal gestational weight gain [45, 47].

## Cardiovascular and metabolic development in childhood

Maternal prepregnancy obesity and excessive gestational weight gain are associated with an increased risk of obesity in childhood and adolescence (Table 2). A meta-analysis among 4 studies showed that maternal prepregnancy obesity was associated with a threefold higher risk of childhood obesity [60]. Similarly, a meta-analysis among 12 studies showed that as compared to a recommended amount of gestational weight gain according to the IOM criteria, excessive gestational weight gain was associated with a 33 % increased risk of childhood obesity [61]. The associations of maternal obesity during pregnancy with more detailed childhood fat mass measures have also been studied. Several studies have shown that a higher maternal prepregnancy body mass index is associated with a higher childhood waist circumference and total body fat mass [62–65]. Result from the Generation R Study in Rotterdam, The Netherlands, have previously shown among 4871 mother-offspring pairs that a higher maternal prepregnancy body mass index was associated with a higher childhood total body fat mass, android/gynoid fat mass ratio, abdominal subcutaneous fat mass and abdominal preperitoneal fat mass, a measure of visceral fat mass [62]. Higher maternal weight gain during pregnancy is, independent from maternal prepregnancy body mass index, associated with a higher childhood body mass index, but associations with other fat mass measures are less consistent [65–70]. Also, both maternal prepregnancy obesity and excessive gestational weight gain seem to be associated with a higher blood pressure, adverse lipid profile, insulin resistance and higher inflammatory markers in childhood [62, 66, 68, 71, 72]. However, these associations are less consistent than those for childhood adiposity measures and, if present, seem to be largely mediated by childhood body mass index. Importantly, the associations of maternal body mass index and gestational weight gain with childhood outcomes seem not to be restricted to maternal obesity or excessive gestational weight gain, but are present across the full-range of maternal body mass index and gestational weight gain.

Several studies aimed to identify critical periods of maternal weight during pregnancy for childhood outcomes. A study performed among 5154 UK mother-offspring pairs showed that especially gestational weight gain in the first 14 weeks of pregnancy was positively associated with offspring adiposity at 9 years of age [68]. In line with these findings, a study among 5908 Dutch mother-offspring pairs showed that independent from maternal prepregnancy weight and weight gain in later pregnancy, early-pregnancy weight gain was associated with an adverse cardio-metabolic profile in childhood [66]. A study among 977 mother–child pairs from Greece showed that maternal first trimester weight gain was associated with an increased risk of childhood obesity and a higher childhood diastolic blood pressure [46]. A Finnish study among 6637 mothers and their adolescent offspring showed that maternal weight gain of >7 kg in the first 20 weeks of gestation was associated with the risk of offspring overweight at the age of 16 years [73]. These studies suggest that especially maternal weight gain in early pregnancy, when maternal fat accumulation forms a relatively large component of gestational weight gain, may be a critical period for an adverse childhood cardiovascular risk profile.

Thus, next to the risks of adverse fetal outcomes, maternal prepregnancy obesity and excessive gestational weight gain may lead to increased risks of adiposity and adverse cardiovascular risk factors in childhood and adolescence.

## Cardiovascular and metabolic disease in adulthood

Multiple studies have shown that a higher maternal prepregnancy body mass index is associated with a higher adult body mass index in the offspring, independent from socio-demographic and lifestyle-related confounding factors [74–76] (Table 2). Similarly, increased maternal weight gain during pregnancy has been associated with higher offspring adiposity levels in adulthood [74, 76–79]. A study among 2432 Australians showed that higher maternal gestational weight gain was, independent from maternal prepregnancy body mass index, associated with a higher body mass index and tended to be associated with a higher systolic blood pressure in the offspring at the age 21 years [79]. A study among 1400 mother-offspring pairs in Jerusalem showed that higher maternal prepregnancy body mass index was associated with a higher body mass index, waist circumference, blood pressures, insulin and triglyceride levels and lower HDL cholesterol in the offspring aged 32 years [74]. Adjustment for adult concurrent body mass index fully explained the associations of maternal prepregnancy body mass index with cardio-metabolic risk factors in adulthood. In the same study, higher maternal gestational weight gain was only associated with increased adiposity levels in adult offspring [74]. Another study among 308 Danish mother-offspring pairs, which assessed the associations of maternal weight gain among normal weight women, showed that a higher maternal weight gain was associated with higher insulin levels and leptin levels among male offspring only [77]. Recently, a study using birth records from 37,709 participants, showed that a higher maternal body mass index at the first antenatal visit was associated with an increased risk of premature all-cause mortality and hospital admissions for cardiovascular events in adult offspring [80]. These associations were not explained by adjustment for maternal age at delivery, socioeconomic status, sex of offspring, current age, birth weight, gestation at delivery, and gestation at measurement of body mass index. In the adjusted model, the hazard of all-cause mortality in offspring of obese mothers was 1.35 (95 % CI 1.17–1.55), as compared to offspring from mothers with a normal body mass index. In this study, no information on gestational weight gain or offspring body mass index was available [80].

Thus, in line with the associations of maternal prepregnancy obesity and excessive gestational weight gain with childhood outcomes, these adverse maternal exposures are associated with an increased risk of adiposity and cardiovascular and metabolic disease and mortality in the adult offspring.

## Approaches to assess causality for the observed associations

The major limitation of these observational studies is confounding. Various family-based socio-demographic, nutritional, lifestyle related and genetic characteristics may explain the observed associations of maternal prepregnancy body mass index and gestational weight gain with adverse health outcomes in the offspring. Few studies used more sophisticated study designs to obtain further insight into the role of confounding in the observed associations, including sibling comparison studies, maternal and paternal offspring comparisons analyses, Mendelian randomization studies, and randomized controlled trial analyses, as described previously [38].

First, sibling comparison studies enable better control for potential confounding factors shared within families [81]. A sibling comparison study among offspring from mothers who had high levels of prepregnancy weight loss due to gastrointestinal bypass surgery showed that the risk of overweight and obesity and adverse cardio-metabolic risk factors was higher in children born to mothers before surgery than those born to mothers after surgery [82, 83]. A sibling comparison study among 42,133 women who had more than one singleton pregnancy and their 91,045 offspring showed that higher maternal total gestational weight gain was associated with a higher body mass index in childhood [84]. A study using a sibling comparison design among 280.866 singleton-born Swedish men showed that a higher maternal body mass index in early pregnancy was associated with higher offspring body mass index at the age of 18 years in the whole cohort and between non-siblings, but not within-siblings, which suggests that the association may be explained by confounding environmental characteristics [85]. However, among the same study population it was shown that among overweight and obese mothers, higher total gestational weight gain was associated with higher offspring body mass index at the age of 18 years among siblings, which suggests a possible intra-uterine effect for gestational weight gain [86]. A limitation of sibling comparison studies is that next to the major exposure of interest, such as maternal body mass index, other related characteristics may also change over time.

Second, several studies compared the strength of associations of maternal and paternal body mass index with offspring outcomes as an aid to further disentangle underlying mechanisms. Stronger associations for maternal body mass index suggest direct intrauterine mechanisms, whereas similar or stronger associations for paternal body mass index suggest a role for shared family-based, lifestyle-related characteristics or genetic factors [87]. Studies comparing associations of maternal and paternal body mass index with childhood body mass index have shown conflicting results [88]. However, studies examining these associations with more detailed childhood fat mass measures and other cardio-metabolic risk factors, have shown that maternal prepregnancy body mass index tends to be more strongly associated with childhood total fat mass, android/gynoid fat mass ratio and clustering of cardio-metabolic risk factors than paternal body mass index [62–64]. These findings suggest that some of the effects of maternal prepregnancy obesity on offspring outcomes may be through direct intra-uterine mechanisms.

Third, Mendelian randomization approaches use genetic variants, known to be robustly associated with the exposure of interest and not affected by confounding, as an instrumental variable for a specific exposure [89]. Associations of these genetic variants with the outcomes of interest support causality for these associations. A study among 4091 mother-offspring pairs, showed no association of maternal FTO with childhood fat mass at the age of 9 years [64]. Thus far, no other Mendelian randomization studies on these specific associations have been performed.

Fourth, randomized controlled trials are considered as the golden standard for causality studies. Because randomized studies are difficult to perform when maternal prepregnancy obesity and excessive gestational weight gain are the major exposures of interest, previous studies focused on influencing determinants of obesity and excessive gestational weight gain, such as dietary factors and physical activity levels [48]. Based on results from randomized controlled trials, it has been suggested that especially dietary interventions during pregnancy may lead to a small reduction in the amount of gestational weight gain [48]. However, whether they also have a beneficial effect on long-term offspring health outcomes remains unclear. A small randomized controlled trial among 254 mothers and their children, which provided both dietary advice and exercise to obese women during pregnancy, observed no difference in body mass index or metabolic risk factors in the offspring at the age of 2.8 years, when compared to the control group and an external reference group of normal weight women [90].

Altogether, results from studies specifically designed to explore the causality for the associations of maternal obesity during pregnancy with offspring outcomes are not conclusive yet.

## Programming effects of maternal obesity during pregnancy

### Hypothesis

**Fig. 1:**
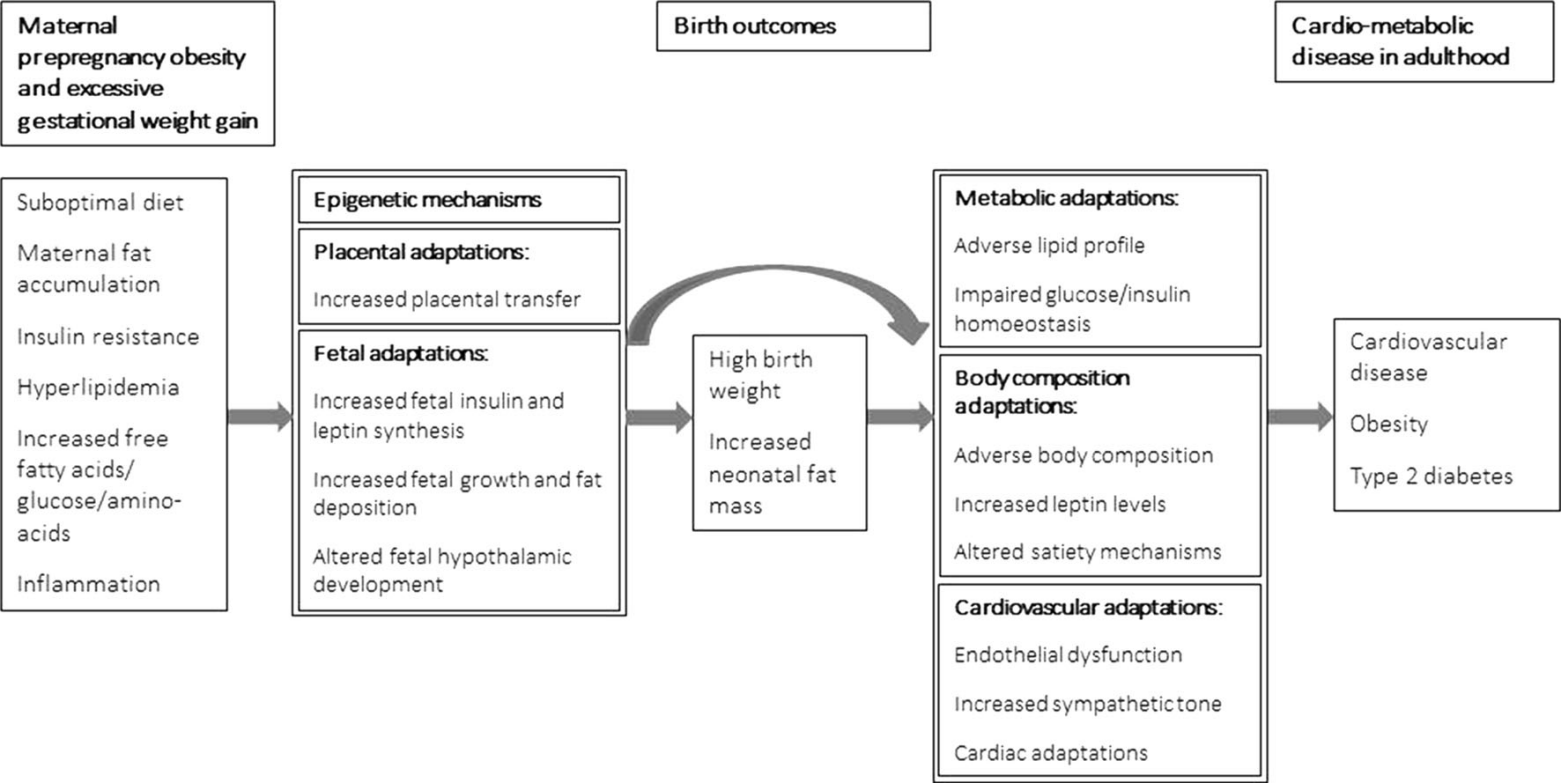
Maternal obesity during pregnancy and offspring developmental adaptations (conceptual model for potential underlying mechanisms for the associations of maternal obesity during pregnancy with adverse cardiovascular and metabolic health outcomes in offspring)

The mechanisms underlying the associations of maternal obesity or excessive gestational weight gain with cardiovascular and metabolic disease in the offspring are not known yet. The fetal overnutrition hypothesis suggests that increased placental transfer of nutrients to the developing fetus in obese mothers and mothers with high levels of gestational weight gain, may subsequently affect fetal development, fetal fat deposition and the development of the hypothalamic-endocrine system that controls appetite and energy metabolism [91–93]. These adaptations may predispose individuals to a greater risk of adverse health outcomes in later life. Figure 1 shows potential mechanisms that might be involved in the associations of higher maternal prepregnancy body mass index and gestational weight gain with the risks of cardiovascular and metabolic disease in the offspring.

### Specific maternal exposures

Maternal prepregnancy obesity and excessive gestational weight gain are complex traits, which reflect multiple components. Maternal prepregnancy obesity reflects maternal nutritional status, fat accumulation and low-grade inflammation, whereas maternal weight gain during pregnancy additionally reflects maternal and amniotic fluid expansion and growth of the fetus, placenta and uterus [37].

Maternal prepregnancy obesity is an indicator of a poor quality maternal diet. Both macronutrients and micronutrients intake related to a Western diet may influence fetal cardiovascular and metabolic development, through influences on placental transfer and subsequently offspring fat deposition, adipocyte function, pancreatic function and food preference [91, 94]. A study among 585 mothers and their children showed that a maternal diet during pregnancy high in saturated fat and sugar intake was associated with an increased risk of offspring obesity [95]. Also, several studies suggested that a low maternal Omega-3 and high Omega-6 fatty acids intake and plasma levels are associated with an increased risk of obesity in the offspring [96–98]. A study among 906 mother–child pairs showed that higher maternal dietary glycemic index and glycemic load in early pregnancy, but not later in pregnancy, were associated with higher fat mass in childhood [99]. A study among approximately 3000 mothers, fathers and their children showed that maternal dietary intake of proteins, fat and carbohydrates during pregnancy, but not paternal dietary intake, was associated with the child’s dietary intake of the same macronutrients. The associations of maternal dietary intake during pregnancy with child’s dietary intake were also stronger than the associations of maternal postnatal dietary intake, which suggest that in utero mechanisms may play a role in the programming of offspring appetite [100]. Altogether, these studies suggest that various measures reflecting a suboptimal dietary status in pregnant women are associated with adverse cardiovascular and metabolic outcomes in offspring.

Maternal prepregnancy obesity and excessive gestational weight gain partly reflect maternal fat accumulation, which is important for fetal development [101]. However, during pregnancy, fat accumulation predominantly occurs centrally [101]. Central fat accumulation is associated with an adverse cardiovascular and metabolic risk profile in adults, including pregnant women [102]. The metabolic disturbances may involve dyslipidemia and insulin resistance, which leads to higher maternal circulating levels of free fatty acids, amino acids and glucose, which affect placental and fetal development [101]. A small study among 40 pregnant women showed that maternal insulin secretory response in early pregnancy, but not before or later in pregnancy, was associated with increased placental growth [103]. It has been suggested that excessive maternal nutrition to the developing fetus, especially hyperglycemia, may lead to teratogenicity in the first trimester of pregnancy [104, 105]. Also, gestational diabetes, glycosuria and higher maternal fasting glucose levels during pregnancy are associated with higher weight and c-peptide levels at birth and body mass index, fat mass level, fasting glucose and insulin levels in the offspring [106–110]. Two sibling comparison studies, conducted in the Pima of Arizona and Sweden, respectively, showed that body mass index and the risk of type 2 diabetes were higher among offspring from mothers with diabetes during pregnancy, as compared to their siblings who were born when the mother did not have diabetes [85, 111]. Small observational studies have suggested that, independent from maternal prepregnancy body mass index, higher maternal triglyceride and amino acid levels are associated with a higher birth weight and neonatal fat mass [112–115]. Thus, maternal fat distribution and metabolic profile during pregnancy may have persistent effects on fetal development and cardio-metabolic development in later life.

Low grade systemic inflammation may be involved in pathways leading from maternal obesity or excessive gestational weight gain to adverse offspring outcomes. Obesity is associated with low-grade systemic inflammation and oxidative stress, also during pregnancy [116–118]. Additionally, pregnancy itself leads to a state of mild maternal systemic inflammation, which may interact with obesity-mediated inflammatory mechanisms [119–122]. It has been shown that maternal inflammatory markers during pregnancy correlate with fetal growth and neonatal fat mass [123, 124], but the effects at older ages are less clear. A recent study among 439 Danish mother-offspring pairs, observed no associations of maternal third trimester CRP, TNF-α, IL-6, and IL-1β with offspring body mass index, waist circumference, blood pressure and several metabolic measures at the age of 20 years [125].

### Underlying pathways

Both maternal obesity and excessive gestational weight gain as well as the correlated nutritional, body fat distribution, metabolic and inflammatory exposures may lead to programming effects in the offspring through several pathways.

Epigenetic changes in the offspring are likely to play an important role in developmental programming [126]. Epigenetic mechanisms involve a range of modifications to DNA and associated proteins that together regulate gene activity [127]. Environmental influences in early life may induce epigenetic changes, and thereby affect the risk of cardiovascular and metabolic disease in later life [127]. Although animals studies provide support for epigenetic modifications due to maternal obesity or a high fat diet, only few human studies have explored these associations [126]. Small studies among pregnant women suggested epigenetic changes of placental genes induced by maternal obesity and impaired maternal glucose tolerance [121, 128–130]. Also, a human study among 88 mother–child pairs suggested that maternal weight gain in early pregnancy, but not maternal prepregnancy body mass index or weight gain in later pregnancy, might be associated with epigenetic modifications in offspring cord blood [131]. Epigenetic modifications as well as other mechanisms may be involved in adiposity, cardiovascular and metabolic developmental adaptations.

Offspring from mothers with prepregnancy obesity or excessive gestational weight gain during pregnancy have higher fetal growth rates and are at increased risk of being born large for their gestational age. Higher birth weight is associated with an increased risk of obesity in later life [132]. The associations of maternal obesity during pregnancy with the risk of obesity in childhood and adulthood may thus be explained by tracking of body size and fatness throughout the life course. However, many observational studies have shown that additional adjustment for birth weight does not explain the observed associations [38]. This might be explained by birth weight not accurately reflecting neonatal fat mass, but might also suggest that other mechanisms play an important role [91]. Animal studies have suggested that altered adipocyte development may influence the development of obesity and insulin resistance in the offspring, as maternal obesity during pregnancy may affect both offspring adipocyte morphology and metabolism [92]. Next to altered growth and adipocyte function, altered appetite control may be a key factor in developmental programming of obesity. A maternal hypercalorific diet during pregnancy and overfeeding in the fetal and early postnatal period is associated with adverse programming of the hypothalamus, which may lead to hyperphagia and altered satiety mechanisms [91, 133]. High leptin and insulin levels in the fetal and early postnatal period are thought to play a central role in this adverse hypothalamic programming [91].

The associations of maternal prepregnancy body mass index and gestational weight gain with adverse cardiovascular and metabolic outcomes in the offspring appear to be largely mediated through offspring obesity. However, direct cardiovascular and metabolic programming effects of maternal obesity during pregnancy may also be present [92]. Animal studies have shown maternal programming effects on cardiovascular and metabolic outcomes. Maternal obesity and high fat diet during pregnancy are associated with high blood pressure, endothelial dysfunction, increased aortic stiffness and cardiac hypertrophy in rodents [92, 134]. It has been shown that high blood pressure in offspring occurs, due to selective leptin resistance which increased sympathetic nervous system activity, before the development of increased adiposity levels and hyperleptinaemea, which suggests that the selective leptin resistance was not obesity related but a direct consequence of the exposure to maternal obesity in early life [134]. Increased maternal glucose transport to the developing fetus leads to a higher exposure of glucose for the developing fetal pancreas, which results in acceleration of fetal pancreatic development [92, 133]. This accelerated pancreatic maturation may predispose to premature loss of ß-cells and consequently lead to impaired glucose and insulin homoeostatis [133]. Offspring of female rats and mice fed a high-fat diet during pregnancy have increased triglyceride levels and oxidative stress in the liver, which may predispose to the development of non-alcohol fatty liver disease, the hepatic manifestation of the metabolic syndrome [92].

Thus, multiple mechanisms may be involved in the intra-uterine pathways leading from maternal obesity and excessive weight gain during pregnancy to long-term adverse offspring health outcomes.

## Perspectives for future epidemiological research

Current evidence from epidemiological studies suggests that maternal obesity and excessive weight gain during pregnancy have important adverse consequences on cardiovascular and metabolic development from fetal life onwards, leading to disease in later life. Yet, there remain important issues to be addressed. These include identification of the extent of causality of the observed associations, the underlying exposures and their critical periods, the developmental adaptations, and the potential for development of preventive strategies (Table 3).

**Table 3:** Key points for future research

Observational studies with a sophisticated study design, such as parent-offspring comparison studies, sibling comparison studies and Mendelian randomization-studies, are needed to obtain further insight into the causality of the observed associations
Detailed maternal exposures and offspring outcome measurements are needed to obtain further insight into the exposures and their critical periods, and the underlying mechanisms of the observed associations
Long-term follow-up of participants in trials focused on reducing maternal weight throughout pregnancy is needed for assessment of causality of the observed associations and the effectiveness of maternal lifestyle interventions during pregnancy for improving long-term health of offspring

First, despite extensive adjustment for potential confounding factors in these observational studies, residual confounding may still be an issue. The causality of the observed associations needs to be further addressed. Large observational studies that are able to apply more sophisticated methods, such as parent-offspring comparison studies, sibling comparison studies and Mendelian randomization-studies are needed to obtain further insight into the causality of the associations of interest. Long-term follow-up of participants in trials focused on reducing maternal weight throughout pregnancy will also provide further insight into the causality. Large meta-analyses are needed to obtain further insight into the strength, consistency and independency of the associations.

Second, the underlying mechanisms of the observed associations of maternal obesity during pregnancy with offspring health outcomes remain unclear. Animals studies have identified multiple pathways that may be involved in these associations, but these pathways remain largely unexplored in humans. Maternal prepregnancy obesity and excessive gestational weight gain are complex traits, which reflect multiple lifestyle-related and biological components, which complicates identification of potential underlying pathways. Future studies may benefit from detailed assessments of the studied exposures and outcomes throughout the life-course, which could provide further insight into potential underlying mechanisms. Studies with repeated maternal weight measurements during pregnancy available can identify critical periods of maternal weight gain. To obtain further insight into the different components associated with offspring outcomes, studies are needed with more detailed measurements of maternal nutritional status, body composition, metabolic and inflammatory measures, pregnancy-related hemodynamic adaptations and fetal growth. For the offspring outcomes, more detailed measurements of growth, body composition and cardio-metabolic factors, including cardiac structures, endothelial function, pulse wave velocity, lipid spectrums and glucose responses, might also lead to further insight into the underlying growth, vascular and metabolic mechanisms present in the observed associations. Since early pregnancy appears to be a critical period for offspring outcomes, studies are needed with detailed maternal measurements from early pregnancy onwards to already assess their influence on placental and embryonic growth and development. Long-term follow up of participants in observational studies is needed to assess the influence on the risk of obesity and related cardio-metabolic disorders throughout the life-course. In addition, it is of interest to conduct follow-up studies of the third generation offspring from these large observational studies, as this may provide further insight into the intergenerational effects of maternal obesity during pregnancy on especially female offspring.

Third, further research is needed focused on prevention of adverse health outcomes in offspring through optimizing maternal prepregnancy body mass index, weight gain and diet during pregnancy. Studies are needed to assess the optimal amounts of maternal weight gain for short-term and long-term maternal and offspring health outcomes to further improve the IOM recommendations for gestational weight gain. Identification of specific maternal dietary components associated with offspring health outcomes is needed to improve maternal dietary recommendations during pregnancy. Long term follow up of mothers and their children participating in randomized trials focused on improving maternal diet and reducing maternal weight throughout pregnancy will provide insight into the effectiveness of these maternal lifestyle interventions during pregnancy for improving long-term health of offspring.

## Conclusions

Maternal prepregnancy obesity and excessive weight gain during pregnancy seem to be important risk factors for an adverse in utero environment and long-term adverse cardiovascular and metabolic outcomes in the offspring. Well-designed epidemiological studies are needed to identify the extent of causality of the observed associations, the underlying exposures and their critical periods, the developmental adaptations, and the potential for development of preventive strategies to improve long-term health outcomes of offspring.
